# The complete chloroplast genome of *Agave angustifolia*

**DOI:** 10.1080/23802359.2021.1941360

**Published:** 2021-10-22

**Authors:** Xu Qin, Xinli Yang, Xing Huang, Gan Jin, Xiangyan Yang, Mi Wu, Tao Chen, Kexian Yi

**Affiliations:** a Guangxi Subtropical Crops Research Institute, Nanning, P. R. China; b College of Tropical Crops, Hainan University, Haikou, P. R. China; c Environment and Plant Protection Institute, Chinese Academy of Tropical Agricultural Sciences, Haikou, P. R. China

**Keywords:** *Agave angustifolia*, chloroplast genome, phylogenetic tree

## Abstract

*Agave angustifolia* is commonly used for the production of bacanora, a kind of fermented and distilled beverage in Mexico. In the present study, we have successfully assembled its chloroplast genome. The full length of the genome is 157,274 bp with the GC content of 37.84%. There is a large single copy region (LSC) of 85,895 bp, a pair of inverted repeat regions (IR) of 26,575 bp and a small single copy region (SSC) of 18,229 bp in the genome. A total of 132 genes are annotated in the cp genome. Among these, there are 86 protein-coding genes, 38 tRNAs and 8 rRNAs. Phylogenetic analysis reveals that *A. angustifolia* is closely related with *A*. H11648.

*Agave angustifolia* is a common surface plant ranged from Mexico to Panama, with remarkable adaptation to drought, heat and cold environments (Garca-Mendoza and Chiang 2003). It is commonly used for the production of bacanora, a kind of fermented and distilled beverage (Gutiérrez-Coronado et al. 2007). The leaf fiber of *A. angustifolia* has a high cellulose content (67%), which contributes to the widely cultivation of its hybrid progeny *A.* H11648 around the world (Robert et al. 2008; Rosli et al. 2013). There exists a certain amount of fibrous waste after beverage or fiber production of *Agave* plants, which could be used as feedstock for biofuels production (Flores-Gómez et al. 2018). Till now, the genomic basis of chloroplast (cp) is still weak in *A. angustifolia*, even if leaf is the main above ground organ for vegetative growth and photosynthesis. A previous study has reported that *A. angustifolia* was not closely related with *A.* H11648 (Huang et al. 2018). Thus, we assembled the complete cp genome of *A. angustifolia* by Illumina sequencing, in order to reveal its systematic position at cp genome level, which could also lay the foundation for future studies related to chloroplast of *A. angustifolia.*

The leaves of *A. angustifolia* were collected at the germplasm garden (22.90°N, 108.33°E) of Guangxi Subtropical Crops Research Institute, Nanning, China. The total genomic DNA was isolated with the modified CTAB method (Doyle and Doyle 1987). The specimen was deposited in Herbarium of Guangxi Subtropical Crops Research Institute (HGS-jm2020011). DNA sample was sent to Biozeron Biotech (Shanghai, China) for library construction and sequencing. A total of 6.34 Gb raw data was generated by Illumina NovaSeq platform and then deposited to SRA under the accession of PRJNA705379. NOVOPlasty software was used for cp genome assembly, which was then gap filled by GapCloser software (Luo et al. 2012; Dierckxsens et al. 2017). The cp genome was annotated and corrected by DOGMA and Geneiousv11.0.3, respectively (Wyman et al. 2004; Kearse et al. 2012). The full cp genome sequence of *A. angustifolia* was deposited to GenBank with the accession number MW540498.

The total length of *A. angustifolia* cp genome is 157,274 with the GC content of 37.84%. There is a large single copy region (LSC) of 85,895 bp, a pair of inverted repeat regions (IR) of 26,575 bp and a small single copy region (SSC) of 18,229 bp in the genome. A total of 132 genes are annotated in the cp genome. Among these, there are 86 protein-coding genes, 38 tRNAs and 8 rRNAs.

A total of 27 cp genome sequences were selected for phylogenetic analysis, including 24 species in Agavoideae and 3 other species as outgroup (*Albuca kirkii*, *Nolina atopocarpa* and *Oziroe biflora*) (McKain et al. 2016; Lee et al. 2019; Jin et al. 2020). The MAFFT software was used for the alignment of those long sequences (Katoh and Standley 2013). We constructed a Maximum Likelihood phylogenetic tree with 1000 bootstrap replicates by MEGA7 software (Kumar et al. 2016). The result revealed that *A. angustifolia* is closely related with *A*. H11648 (Figure 1), which indicated the genetic relationship between the two species. This study would expand the number of plant chloroplast genomes and benefit relevant studies of chloroplast in agave species.

**Figure 1. Phylogenetic tree of 27 chloroplast genomes.**
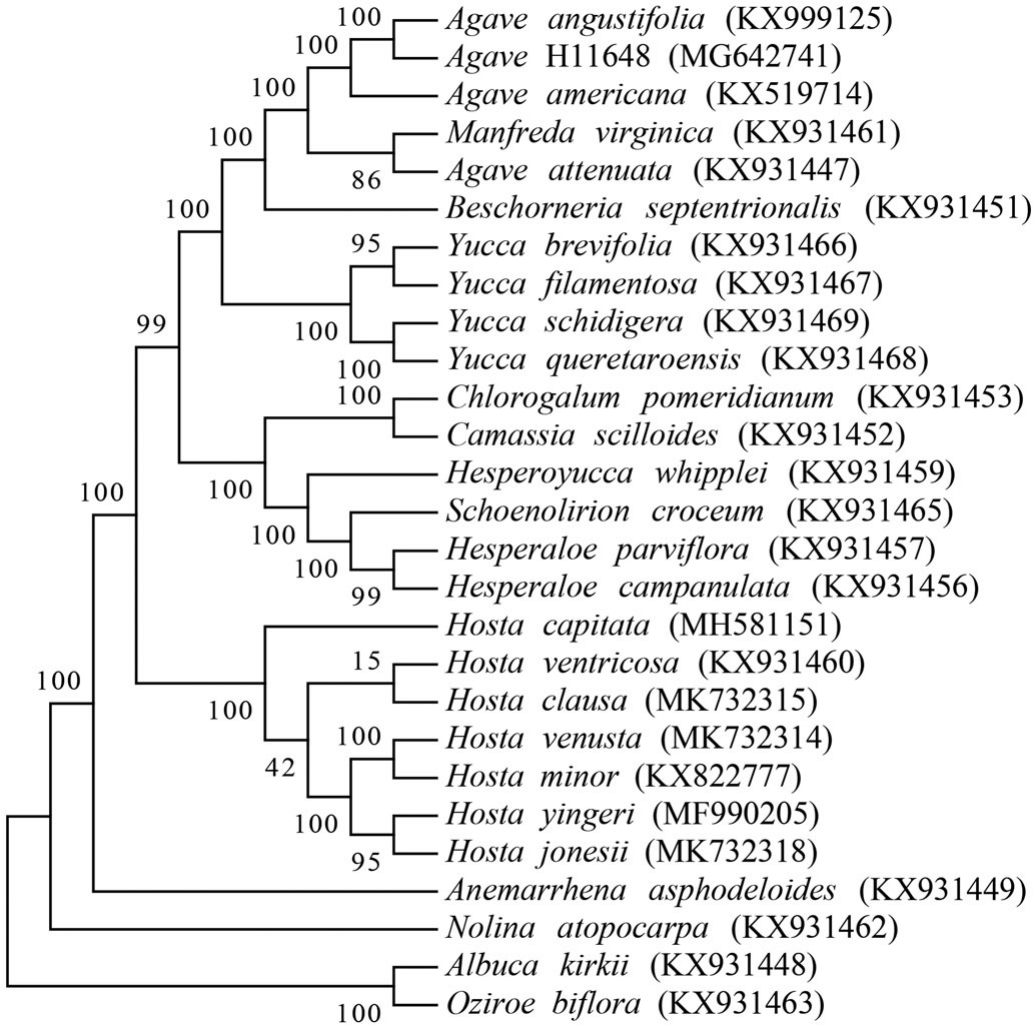


## Data Availability

The data that support the findings of this study are fully available in SRA (https://www.ncbi.nlm.nih.gov/sra/?term=PRJNA705379) and GenBank (https://www.ncbi.nlm.nih.gov/nuccore/MW540498).

## References

[CIT0001] Dierckxsens N, Mardulyn P, Smits G. 2017. NOVOPlasty: de novo assembly of organelle genomes from whole genome data. Nucleic Acids Res. 45:e18. 28204566 10.1093/nar/gkw955 PMC5389512

[CIT0002] Doyle JJ, Doyle JL. 1987. A Rapid DNA isolation procedure from small quantities of fresh leaf tissues. Phytochem Bull. 19:11–15.

[CIT0003] Flores-Gómez CA, Escamilla Silva EM, Zhong C, Dale BE, da Costa Sousa L, Balan V. 2018. Conversion of lignocellulosic agave residues into liquid biofuels using an AFEX™-based biorefinery. Biotechnol Biofuels. 11:7. 29371883 10.1186/s13068-017-0995-6 PMC5769373

[CIT0004] Garca-Mendoza A, Chiang F. 2003. The confusion of *Agave vivipara* L. and *A. angustifolia* Haw., two distinct taxa. Brittonia. 55(1):82–87.

[CIT0005] Gutiérrez-Coronado M, Acedo-Félix E, Valenzuela-Quintanar A. 2007. Bacanora industry and its process of production. CYTA-J Food. 5(5):394–404.

[CIT0006] Huang X, Wang B, Xi J, Zhang Y, He C, Zheng J, Gao J, Chen H, Zhang S, Wu W, et al. 2018. Transcriptome comparison reveals distinct selection patterns in domesticated and wild Agave species, the important CAM plants. Int J Genom. 2018:5716518. 10.1155/2018/5716518 PMC6282153 30596084

[CIT0007] Jin G, Huang X, Chen T, Qin X, Xi J, Yi K. 2020. The complete chloroplast genome of agave hybrid 11648. Mitochondrial DNA B Resour. 5(3):2345–2346. 33457785 10.1080/23802359.2020.1775145 PMC7781945

[CIT0008] Katoh K, Standley DM. 2013. MAFFT multiple sequence alignment software version 7: improvements in performance and usability. Mol Biol Evol. 30(4):772–780. 23329690 10.1093/molbev/mst010 PMC3603318

[CIT0009] Kearse M, Moir R, Wilson A, Stones-Havas S, Cheung M, Sturrock S, Buxton S, Cooper A, Markowitz S, Duran C, et al. 2012. Geneious Basic: an integrated and extendable desktop software platform for the organization and analysis of sequence data. Bioinformatics. 28(12):1647–1649. 22543367 10.1093/bioinformatics/bts199 PMC3371832

[CIT0010] Kumar S, Stecher G, Tamura K. 2016. MEGA7: molecular evolutionary genetics analysis version 7.0 for bigger datasets. Mol Biol Evol. 33(7):1870–1874. 27004904 10.1093/molbev/msw054 PMC8210823

[CIT0011] Lee SR, Kim K, Lee BY, Lim CE. 2019. Complete chloroplast genomes of all six Hosta species occurring in Korea: molecular structures, comparative, and phylogenetic analyses. BMC Genomics. 20(1):833. 31706273 10.1186/s12864-019-6215-y PMC6842461

[CIT0012] Luo R, Liu B, Xie Y, Li Z, Huang W, Yuan J, He G, Chen Y, Pan Q, Liu Y, et al. 2012. Soapdenovo2: an empirically improved memory-efficient short-read de novo assembler. Gigascience. 1(1):18. 23587118 10.1186/2047-217X-1-18 PMC3626529

[CIT0013] McKain MR, McNeal JR, Kellar PR, Eguiarte LE, Pires JC, Leebens-Mack J. 2016. Timing of rapid diversification and convergent origins of active pollination within Agavoideae (Asparagaceae). Am J Bot. 103(10):1717–1729. 27793858 10.3732/ajb.1600198

[CIT0014] Robert ML, Lim KY, Hanson L, Sanchez-Teyer F, Bennett MD, Leitch AR, Leitch IJ. 2008. Wild and agronomically important *Agave* species (Asparagaceae) show proportional increases in chromosome number, genome size, and genetic markers with increasing ploidy. Bot J Linn Soc. 158(2):215–222.

[CIT0015] Rosli NA, Ahmad I, Abdullah I. 2013. Isolation and characterization of cellulose nanocrystals from *Agave angustifolia* fibre. Bioresources. 8(2):1893–1908.

[CIT0016] Wyman SK, Jansen RK, Boore JL. 2004. Automatic annotation of organellar genomes with dogma. Bioinformatics. 20(17):3252–3255. 15180927 10.1093/bioinformatics/bth352

